# Investigating a Curcumin-Loaded PLGA-PEG-PLGA Thermo-Sensitive Hydrogel for the Prevention of Alzheimer’s Disease

**DOI:** 10.3390/antiox11040727

**Published:** 2022-04-07

**Authors:** Yi-Wen Lin, Chih-Hsiang Fang, Ching-Yun Yang, Ya-Jyun Liang, Feng-Huei Lin

**Affiliations:** 1 Institute of Biomedical Engineering, National Taiwan University, No.1, Sec. 1, Jen-Ai Road, Taipei 10617, Taiwan; d06548012@ntu.edu.tw (Y.-W.L.); r06548013@ntu.edu.tw (C.-Y.Y.); d04548017@ntu.edu.tw (Y.-J.L.); 2 Trauma and Emergency Center, China Medical University Hospital, No.2, Xueshi Rd., North Dist., Taichung 40454, Taiwan; d04548007@ntu.edu.tw; 3 Division of Biomedical Engineering and Nanomedicine Research, National Health Research Institutes, No. 35, Keyan Road, Zhunan 35053, Taiwan

**Keywords:** Alzheimer’s disease, curcumin, amyloid beta, poly(lactic-co-glycolic acid), poly(ethylene glycol)

## Abstract

In Alzheimer’s disease (AD), the most common cause of dementia, patients generally forget to take pills or skip medication due to side effects, affecting the treatment efficacy. In this study, we combined a poly(lactic-co-glycolic acid), (PLGA)-poly(ethylene glycol), and (PEG)-PLGA thermo-sensitive hydrogel with curcumin (PGC) to deliver an intramuscular injection that could continuously release curcumin and maintain it at a constant level in blood to prevent AD development or progression. We evaluated the drug release profile and cytotoxicity of PGC and its effects on AD pathology through in vitro and in vivo studies and on cognitive function through an aluminum-chloride-induced AD rat model. In the in vitro study, PGC exhibited a lack of cytotoxicity, excellent anti-inflammatory and antioxidant properties, and microglial modulation. In the Morris water maze test, the PGC injection-administered AD rats presented well-focused searching behavior with the shortest swimming path and longest retention times in the quadrant where the platform was initially located. Furthermore, PGC reduced amyloid-beta aggregation and deposition and significantly increased hippocampal activity. This study demonstrated that intramuscular PGC injection can effectively prevent AD development or progression in rats without inducing toxicity; therefore, this strategy could help overcome the present challenges in AD management in humans.

## 1. Introduction

Dementia involves a decline in cognitive function beyond what is expected in normal aging; it is usually chronic and progressive. It affects memory, thinking, orientation, comprehension, calculation, language, judgment, and learning abilities. Cognitive impairment is often accompanied by, and sometimes ensues from, the deterioration of emotional control, social behavior, or motivation. Acquired deficits in multiple cognitive domains characterize dementia [1]. Alzheimer’s disease (AD) is the most common cause of dementia, accounting for up to 80% of dementia cases. It is an unexplained chronic and irreversible primary neurodegenerative disease of the brain that can lead to insidious paroxysmal dementia, most commonly in later life. AD is characterized by a progressive decline in brain function, especially memory. Brain pathology is characterized by progressive loss of neurological function, along with the appearance of amyloid plaques and neurofibrillary tangles in the brain [2]. The pathogenesis of AD has been gradually deciphered since 1907 [3] when Alois Alzheimer reported the first case, but to date, there is no appropriate treatment to slow the progression of AD.

Treatment of AD can be divided into non-pharmacological therapy and pharmacological therapy. Both approaches aim to improve the quality of life of the patient and maintain cognition and daily activities. Although non-pharmacological treatments such as coconut oil [4], omega-3 fatty acids [5], coenzyme Q10 [6], and coral calcium [7] are safer and less expensive than prescription drugs, their use is not widely supported by health authorities as they lack approved clinical tests and solid scientific evidence. For drug therapy, there are varieties of FDA-approved prescription drugs available for AD treatment including cholinesterase inhibitors and NMDA glutamate receptor antagonists such as Razadyne (galantamine), Exelon (rivastigmine), Aricept (donepezil), and Namenda (memantine). Even though these drugs are effective in AD, they provide only temporary symptom relief, and none have shown the ability to cure or prevent disease progression [8].

Drug treatment can alleviate the symptoms of AD significantly and immediately. However, the side effects of the drugs have always troubled researchers and patients. Side effects include nausea, diarrhea, vomiting, and dizziness [9]. Additionally, patients often forget to take medications because of memory loss or give up medicines because of side effects, affecting the treatment outcomes. Therefore, in this study, we aimed to explore AD prevention, based on the amyloid-β (Aβ) hypothesis, by blocking the stacking of the Aβ peptide into larger structures, thereby precluding the formation of oligomers or fiber structures from causing brain damage.

Curcumin is extracted from the rhizome of Curcuma longa, a common spice in South Asia. It is used to treat various diseases such as respiratory disorders, liver dysfunction, and rheumatism [10]. Curcumin is a small molecule drug that can penetrate the blood–brain barrier and reach the brain effectively; its mechanism of action includes anti-oxidation, anti-inflammatory effects, reduction in Tau protein phosphorylation, and inhibition of Aβ peptide production and accumulation [10,11]. Additionally, the structure of curcumin can effectively prevent Aβ peptide stacking [12]. The structure can effectively bind to the Aβ polypeptide, inhibiting its stacking into larger oligomers. The double bond structure connecting the two benzene rings maintains an appropriate distance between the rings and imparts stability to curcumin, meaning it can stably bind to the Aβ peptide. Copper ions have also been shown to help Aβ peptide stacking and Aβ1-42 production [13], and curcumin has been reported to sequester the copper ions in the body and prevent Aβ peptide aggregation after binding these copper ions [14]. The toxicity of the Aβ peptide is primarily attributed to the combination of metal ions, which produces free radicals that cause cell death, stimulate cells to produce more Aβ1-42, and cause abnormal phosphorylation of Tau proteins [15].

Unfortunately, curcumin is not easily absorbed by the human body and is rapidly degraded [16]. Curcumin is hydrophobic and cannot be directly injected intravenously, limiting its clinical use. Therefore, extensive research has been conducted on developing a drug delivery system to overcome this problem. Drug delivery systems such as micelles, liposomes, and phospholipid complexes protect curcumin from degradation and allow curcumin to dissolve in water for injection [17,18]. In a previous study, a thermo-gel was used to deliver curcumin. This drug delivery system has significant advantages such as high drug loading efficiency, injectability, and an extended drug release profile [19]. Triblock copolymers (poloxamers) composed of poly(ethylene glycol-b-propylene glycol-b-ethylene glycol) exhibit reversible solution–gel (sol–gel) behavior in an aqueous solution; nevertheless, they dissolve in a few days.

**Figure 1:**
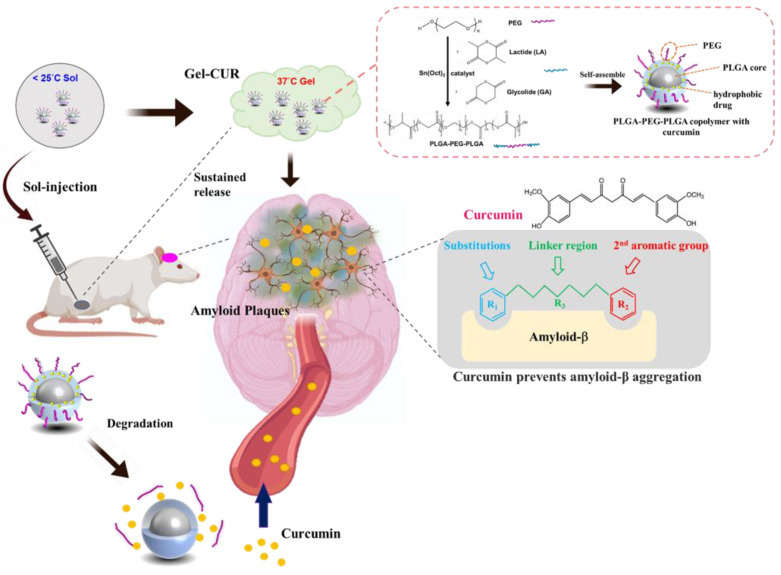
The scheme of the experimental design. This study used the N2a cell line as an in vitro model to investigate the decrease in amyloid-β plaque by a PLGA-PEG-PLGA thermo-sensitive hydrogel with curcumin (PGC) for intramuscular injection. This was to achieve continuous release of curcumin and maintain a constant level of curcumin in the blood, thus preventing Alzheimer’s disease (AD) development or progression. In addition to the in vitro model, an AD rat model was induced by intraperitoneal injection of AlCl_3_ three times a week. The Morris water maze and functional MRI were then used to assess the rats’ working and spatial memory retention. Finally, a biochemical analysis was used to evaluate the effect of AD prevention by PGC.

In this study, poly(lactic-co-glycolic acid) (PLGA) and poly(ethylene glycol) (PEG) were used to prepare a PLGA-PEG-PLGA sol–gel aqueous solution, which has been approved by the FDA due to its excellent biocompatibility. PLGA-PEG-PLGA forms micelles and separates in an aqueous solution at a low temperature [20]. The micelles enhance not only the solubility of curcumin but also the sol–gel properties. PLGA-PEG-PLGA sol–gel behavior is attributed to the intermolecular force between the PEG of the micelles at the appropriate temperature, at which point it transforms from a solution to a gel. The PLGA-PEG-PLGA sol–gel system has been used to deliver curcumin and enhance curcumin solubility. It is designed to transform from a free-flowing solution to a gel at 37 °C. It was the focus of this study because it additionally enables long-term prevention of AD through the controlled release of curcumin, which could reduce oxidative stress, inflammation, and Aβ1-42 production. We aimed to combine curcumin with a carrier to deliver one shot of intramuscular (IM) injection a month in outpatient clinics, which may avoid skipping daily medications by patients and maintain a constant blood level of curcumin through continuous-release, overcoming the above-mentioned obstacles in AD management. The graphic abstract is shown in Figure 1, and this was the first study using the PLGA sol–gel system to encapsulate curcumin for the prevention of AD. For the in vitro studies, cytotoxicity, ThT staining, and biochemical analysis were used to investigate the biocompatibility, β-secretase inhibition, and cellular reactive oxygen species generation of PGC. For the animal study, an AD rat model was induced by intraperitoneal injection of AlCl_3_; the Morris water maze and functional MRI were then used to assess the rats’ working and spatial memory retention.

## 2. Materials and Methods

### 2.1. Materials

Polyethylene glycol-1500 (PEG1500), polyethylene glycol-1000 (PEG1000), d,l-lactide, stannous 2-ethylhexanoate (SnOct2), and glycolide were obtained from Sigma-Aldrich (St. Louis, MO, USA).

### 2.2. Synthesis of PLGA-PEG-PLGA Triblock Copolymer

The PLGA-PEG-PLGA triblock copolymer was synthesized using the ring-opening method. PEG1500 (6 g) was loaded into a three-neck bottle and heated for 2 h at 140 °C in a vacuum. Next, 10 g of d,l-lactide and 2 g of glycolide were added to the bottle at 25 °C. The mixture was heated to 120 °C under vacuum for 15 min. SnOct_2_ (0.04 g) was added as a catalyst and heated to 150 °C under nitrogen for 8 h. After the reaction, the crude polymer was poured into 4 °C ddH_2_O. After complete dissolution, the solution was heated to 80 °C to precipitate the polymer, and the supernatant was removed. The purification was repeated three times, followed by freeze-drying.

### 2.3. Preparation of PLGA-PEG-PLGA Micelles

PLGA-PEG-PLGA (0.4 g) and varying concentrations of curcumin (1, 10, and 25 μM) were dissolved in acetone. The solution was added to a round flask, and the solvent was evaporated using a rotary evaporator (Heidolph, Schwabach, Germany). After the solvent was completely evaporated, 2 mL of ddH_2_O was added to the flask. The solution was sonicated until the copolymer dissolved completely.

### 2.4. Preparation of Aβ Fibrils

Aβ42 peptides were purchased from Peptide Institute, Inc. (ASIA BIOSCIENCE CO. LTD., Taipei, Taiwan). The purchased Aβ42 peptides were dissolved in hexafluoro-2-propanol (HFIP, Oakwood Products, Estill, SC, USA) for monomerization at a final concentration of 1 mM and added to an Eppendorf tube. HFIP was evaporated at room temperature and then stored at −80 °C for later use. Immediately before use, the monomerized Aβ42 peptides in the Eppendorf tube were completely resuspended in 5 mM anhydrous dimethyl sulfoxide (DMSO, catalog number D-2650, Sigma, Allentown, PA, USA) by mixing it using a pipette, and diluted to 100 μM with DMEM (Dulbecco’s modified Eagle’s medium, Sigma, Allentown, PA, USA). The solution was homogenized in a shaker at 37 °C for seven days to aggregate amyloid-β fibrils; the final concentration of amyloid-β fibrils was 100 μM.

### 2.5. Characterization of Triblock Copolymer

#### 2.5.1. ^1^H Nuclear Magnetic Resonance Spectrum of Triblock Copolymer

^1^H Nuclear magnetic resonance (NMR) spectroscopy was used for copolymer structure analysis. The spectra were recorded at 600 MHz on a Bruker 600 MHz spectrometer (AVIII-600MHz, Bruker, Billerica, MA, USA). PLGA-PEG-PLGA was dissolved in CDCl_3_. The d,l-lactide and glycolide ratio of the copolymer was determined using ^1^H NMR.

#### 2.5.2. Fourier Transform Infrared Analysis of Triblock Copolymer

Fourier transform infrared (FTIR) spectra of the synthesized materials including PEG and PLGA-PEG-PLGA were recorded using a FTIR spectrophotometer (Spectrum 100 FTIR Spectrometer, PerkinElmer, Waltham, MA, USA) at the wavenumber range of 450 to 4000 cm^−1^.

#### 2.5.3. Identification of Molecular Weight

The average molecular weight (Mw) and polydispersity index (PDI, Mw/Mn) of the polymers were determined by gel permeation chromatography (GPC 270, Viscotek, Malvern, UK) coupled with a refractive index detector. Tetrahydrofuran (THF) was used as an eluent. The molecular weight was calculated using standard polystyrene samples as references.

#### 2.5.4. Dynamic Light Scattering Analysis

The particle size of the curcumin-loaded micelle was determined by dynamic light scattering (DLS). The micelle solution was diluted to 1% and analyzed immediately. Additionally, the micelles without curcumin were characterized.

#### 2.5.5. Gelation Test and Rheological Analysis

The sol–gel temperature was determined using the inversion test. Cold micelle solutions of different concentrations (15%, 20%, 25%, 30%, and 35%) were heated, the temperature increased in steps of 1 °C, and the vial bottle was inverted. If the solution did not flow within 30 s, the temperature was defined as the sol–gel transition temperature. Furthermore, the sol–gel transition temperature was determined using a rheometer (TA Instruments, New Castle, DE, USA). Micelle solutions of different concentrations were transferred to a Couette cell and overlaid on a film. The solution was heated at 1 °C/min and scanned at a fixed angular frequency of 10 rad/s.

#### 2.5.6. Encapsulation Efficiency and Drug Loading Efficiency

Curcumin was dissolved at different concentrations in a mixture of dimethyl sulfoxide (DMSO) and ddH_2_O (DMSO: ddH_2_O = 9:1). The samples were analyzed at an absorbance wavelength of 435 nm using an enzyme-linked immunosorbent assay (ELISA) reader, and a standard curve was constructed. The micelle solution (100 μL) was diluted nine times with DMSO and analyzed at an absorbance wavelength of 435 nm using the ELISA reader (Multiskan FC, Thermo Fisher, Rockville, MD, USA). Encapsulation efficiency (EE) and drug loading (DL) were calculated using the following equations.

EE (%) = Wentrapped/Wtotal drug × 100%(1)

DL (%) = Wentrapped/Wpolymer × 100%(2)

#### 2.5.7. Drug Release Profile

The curcumin release profile was determined using the ELISA reader. Curcumin was dissolved at different concentrations in a mixture of DMSO and ddH_2_O (DMSO: ddH_2_O = 9:1), and a standard curve was constructed. The micelle solution (0.5 mL, 20%) was loaded into a tube and incubated at 37 °C for 15 min. After the solution transformed to gel, 40 mL of pH 7.4 phosphate-buffered saline (PBS) solution was loaded into the tube and incubated at 37 °C. Every day, after vortexing, 1 mL of PBS solution was removed, and 1 mL of fresh PBS solution was added. Every sample was diluted nine times with DMSO solution and the absorbance of 435 nm wavelength light analyzed using the ELISA reader.

#### 2.5.8. Morphology of Aβ Aggregation and Triblock Copolymer Micelle

Transmission electron microscopy (TEM) was used to observe Aβ aggregation and triblock copolymer micelles. Aβ solution (5 μM) was incubated with or without curcumin-loaded micelles (curcumin concentration, 25 μM) for three days. The incubated samples and non-incubated 5 μM Aβ solution were placed on a TEM 200 mesh grid (Bruker Corporation, Billerica, MA, USA) for 1 min. The excess solution was removed by filter paper, followed by incubation with 20 μL of uranyl acetate (UA, 2%) for 1 min. After the filter paper absorbed the staining solution, the morphology of Aβ was observed using TEM.

#### 2.5.9. In Vitro Study

##### Biocompatibility of Thermo-Gel

The biocompatibility of the thermo-gel was evaluated by using the WST-1 assay in the L929 and N2a cell lines (BCRC, Taiwan) according to the ISO 10993-5 standard. An extract medium was prepared by adding a 0.2 g/mL specimen to high-glucose DMEM (Sigma, Allentown, PA, USA) and incubating it at 37 °C for 24 h. Subsequently, the cells were seeded in 96-well plates at a density of 5 × 10^3^ cells/well and incubated at 37 °C for one day. The culture medium was replaced with the extract medium, and the samples and cells were incubated for one to three days. Before the assay, 10 μL of WST-1 reagent was added to each well and incubated for 4 h. After incubation, the plate was placed in a spectrophotometric plate reader (Multiskan FC, Thermo Fisher, Rockville, MD, USA) set to read the absorbance at 450 nm (with a reference filter at 600 nm) to determine the amount of formazan formed. The percentage of cell viability was calculated using Equation (3):

Cell viability (%) = ([OD experiment−OD background] × 100)/([OD control−OD background])(3)

##### Thioflavin T Fluorescence Assay

Thioflavin T (ThT) is a fluorescent dye initially used to stain amyloid fibrils in histological samples. An aliquot of the Aβ stock solution was diluted to 10 µM in PBS, pH 7.4, and incubated at 37 °C, with shaking, in a 0.5 mL Eppendorf PCR tube (Enfield, CT, USA). For the initial PLGA-PEG-PLGA gel with curcumin (PGC) study, Aβ was mixed with various concentrations of PGC to a final Aβ concentration of 10 µM. When ThT binds to β-sheet aggregate structures such as amyloid fibrils, its fluorescence emission changes. Aβ aggregation was measured by periodically removing 30 μL aliquots from the incubated samples and adding them to 2 mL of 5 μM ThT solution (50 mM phosphate buffer, pH 6.5).

##### Western Blot of β-Secretase Inhibition

Aβ oligomer solution was prepared through the following steps. First, Aβ was dissolved in HFIP and dried overnight. Aβ was resolubilized in 20 μL of DMSO and diluted with 980 μL of phenol-red-free DMEM. The final Aβ concentration was 100 μM. The Aβ oligomer solution was prepared by the addition of 100 μM aluminum chloride to the Aβ solution and incubation at 4 °C overnight. N2a cells were cultured in a 6-well plate (10^5^/well) for 24 h, following which the medium was replaced with 1% fetal bovine serum (FBS) DMEM for three days. The medium was replaced with 10% FBS DMEM with 1 μM curcumin micelle solution (PGC1), 10 μM curcumin-loaded micelle solution (PGC10), 25 μM curcumin micelle solution (PGC25), and 10% FBS DMEM without the micelle solution, followed by incubation for 1 h. The Aβ oligomer solution with 25 μM aluminum chloride, 25 μM aluminum chloride with DMEM alone, or DMEM was separately loaded in 6-well plates and incubated for one day. The N2a cells were then lysed in 100 μL of ice-cold RIPA buffer, and protein concentrations were quantified using the Bio-Rad protein assay. Equal amounts were electrophoresed in 10% sodium dodecyl sulfate (SDS)-polyacrylamide gels. Separated proteins were transferred onto nitrocellulose membranes and incubated with the primary monoclonal mouse antibodies against beta-secretase 1 (BACE1) (1:1000) or α-tubulin (1:2000) overnight at 4 °C. Following incubation with the appropriate secondary antibody, the signals were analyzed using a molecular imager gel system (Bio-Rad, Portland, ME, USA).

##### Determination of Cellular Reactive Oxygen Species Generation

The Aβ oligomer solution was prepared as previously described. N2a cells were cultured in a 96-well plate (10^4^/well) for 24 h; the medium was replaced with 1% FBS DMEM, followed by incubation for three days. The medium was subsequently replaced with PGC1, PGC10, PGC25, or 10% FBS DMEM without the micelle solution for 1 h. The Aβ oligomer solution with 25 μM aluminum chloride, 25 μM aluminum chloride with DMEM alone, or DMEM was separately loaded to a 96-well plate and incubated for one day. The medium was replaced with 2′,7′-dichlorofluorescein diacetate (DCFDA), followed by incubation for 1 h. The fluorescence intensity was measured at excitation wavelengths of 495 nm and 525 nm.

##### PGC Inhibition of Aβ-Induced Inflammation

The Aβ oligomer solution was prepared as previously described; however, the Aβ solution lacked aluminum chloride. BV2 cells were cultured in a 16-well plate (5 × 10^4^/well) for 24 h. The medium was replaced with 10% FBS DMEM, PGC1, PGC10, or PGC25, followed by incubation for 1 h. Aβ oligomer solution and DMEM solution were used to replace the medium in the 16-well plate. The medium was collected after 6 h to analyze interleukin (IL)-1β levels and 24 h to analyze IL-6 and tumor necrotic factor (TNF)-α. The levels of each cytokine were measured using an ELISA kit (R&D system, Minneapolis, MN, USA) according to the procedure indicated by the manufacturer.

##### PGC Inhibition of Aβ-Induced Cytotoxicity

The protocol for preparing the Aβ oligomer solution protocol was as previously described. N2a cells were cultured in a 16-well plate (5 × 10^4^/well) for 24 h; the medium was replaced with 1% FBS DMEM, followed by incubation for three days. Subsequently, the medium was replaced with PGC25 and 10% FBS DMEM without the micelle solution for 1 h. The Aβ oligomer solution with 25 μM aluminum chloride or 25 μM aluminum chloride with DMEM alone was separately loaded to a 16-well plate and incubated for three days. Cell cytotoxicity was measured using a live/dead cell staining kit (TAKARA, Minneapolis, MN, USA) according to the procedure indicated by the manufacturer.

#### 2.5.10. In Vivo Study

##### AD Animal Model

Sprague Dawley male rats (8-week-old) were purchased from Bio LASCO Taiwan Co. Ltd. (Yilan, Taiwan). All experiments were performed in compliance with the National Taiwan University College of Medicine Institutional Animal Care and Use Committee (IACUC no. 20130429). We maintained the animals according to the Guide for the Care and Use of Laboratory Animals. The behavioral tests performed were approved by the Animal Ethics Committee of the National Taiwan University Hospital, Taiwan. AlCl_3_ (7446-70-0, Sigma-Aldrich, Allentown, PA, USA) was dissolved in normal saline before injection. AD was induced using AlCl_3_ intraperitoneal (i.p.) injection three times a week on the basis of previous studies [21]. Thirty rats were randomly categorized into the following five groups of six rats for the in vivo study (Table 1).

#### 2.5.11. Morris Water Maze

The Morris water maze (MWM) was used to assess the working and spatial memory retention in the rats [22]. The circular pool was divided into four quadrants, one of which housed an underwater platform. During each training session, the rat was gently placed in the water at a different drop position and allowed to find the underwater platform; if the rat could not find the platform within 2 min, it was guided toward the platform. After reaching the platform, the rat was allowed to stay on the platform for 30 s. Training was provided in each quadrant for four consecutive days. After four sessions, the time the rats reached the escape platform was recorded using EthoVision software XT (version 11.5, Noldus Information Technology, Wageningen, The Netherlands). Retrieval tests were conducted in two phases: working memory and spatial memory [23]. The time (in seconds) it took each rat to reach the platform from its initial position was recorded to assess spatial memory. In contrast, working memory was assessed by determining the time rats spent in the same quadrant (up to 120 s) but without a platform [24].

##### Functional Magnetic Resonance Imaging

Functional magnetic resonance imaging (fMRI) was performed using a Bruker Biospec 7T fMRI (Bruker Corporation, Billerica, MA, USA). Eight weeks after AlCl_3_ administration and after the treatment injection, the rats were gas-anesthetized prior to fMRI. Two types of images were obtained: one with T2-weighted rapid acquisition and relaxation enhancement (RARE) (anatomical MRI images), and the other with the single-shot gradient-echo plane (GRE-EPI) (fMRI images) [25]. In addition, the coronal plane of the center of the hippocampus was photographed, the brightness of the hippocampus was measured using ImageJ, and statistical parameter mapping (SPM) of the hippocampus was performed using MATLAB-SPM.

##### Histological Analysis and Immunohistochemical Staining

The specimens from the respective groups were fixed, bisected, embedded in paraffin, sectioned at a thickness of 5 μm, and placed on the glass slide. The slides were stained with hematoxylin and eosin (H&E), and immunohistochemical (IHC) analysis was performed for the expression of BACE1. Briefly, the paraffin-embedded tissue blocks were cut into 5 μm thickness for staining. After deparaffinization and rehydration, endogenous peroxidases were blocked with 0.1% hydrogen peroxide (Sigma-Aldrich, Allentown, PA, USA) in PBS solution for 10 min. For retrieval, nonspecific background staining was blocked using 20 μg/mL proteinase K (Sigma-Aldrich, Allentown, PA, USA) solution and incubated for 20 min at 37 °C in a humidified chamber. For immunohistochemistry, primary antibodies, TBS with Aβ [C-Terminal] antibody (25524-1-AP, Proteintech, Rosemont, IL, USA), and 1% bovine serum albumin (BSA) (Abcam, Waltham, MA, USA) were added with appropriate dilution to the tissue sections and incubated at 4 °C overnight. After incubation, tissue sections were rinsed with Tris-buffered saline (TBS) containing 0.025% Triton-X 100 using gentle agitation, and the sections were incubated with TBS containing goat anti-rabbit HRP IgG and 1% BSA. Finally, the tissue sections were stained using 3,3′-diaminobenzidine (DAB, Sigma-Aldrich, Allentown, PA, USA) substrate solution.

##### Statistical Analysis

The data used in the figures are expressed as the mean ± standard deviation (SD). Statistical analysis was performed using a one-way ANOVA, where p-values of less than 0.05 were considered statistically significant.

## 3. Results

### 3.1. Morphology of Micelles, Particle Size Identification, Drug Loading Efficiency, and Drug Release Profile

**Figure 2:**
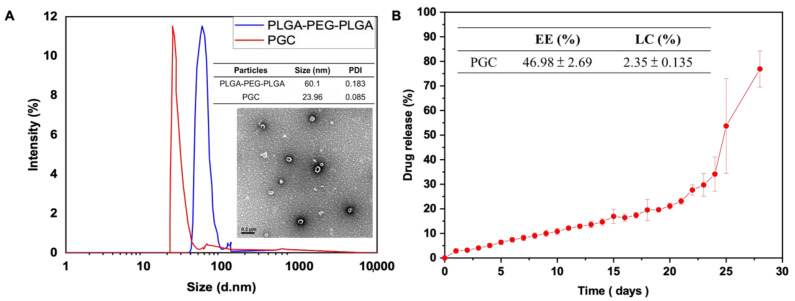
Morphology of micelles, particle size identification, drug loading efficiency, and drug release profile. (**A**) The identification and morphology of particles and (**B**) the loading efficiency and drug release profile.

The particle size of curcumin-loaded micelles and blank micelles is shown in Figure 2A. The size of blank micelles was 60.1 nm and the PDI was 0.183. The size of curcumin-loaded micelles was 23.96 nm and the PDI was 0.085. DLS showed that loading curcumin would decrease the particle size. The particles exhibited a narrow size distribution regardless of the presence of curcumin. TEM images showed spherical nanoparticles, which is consistent with the results obtained by DLS.

The EE and DL were 46.98 ± 2.69% and 2.35 ± 0.135% (*w*/*w*), respectively. The high EE confirmed that this drug delivery system was appropriate for curcumin. We expected that the higher proportion of LA in the copolymer would enable higher EE. The curcumin release profile is shown in Figure 2B. Sustained release from the gel lasted for 20 days. The release from the PLGA-PEG-PLGA system was influenced by hydrogen bonding and intermolecular force [26]. The curcumin released from the gel was dissolved in PBS solution. We estimated that curcumin released from the drug delivery system was loaded in micelles and that the micelles could prevent curcumin degradation in circulation.

### 3.2. The Evaluation of Cell Viability of Thermo-Gel and Inhibition of Aβ-Induced Cytotoxicity

**Figure 3:**
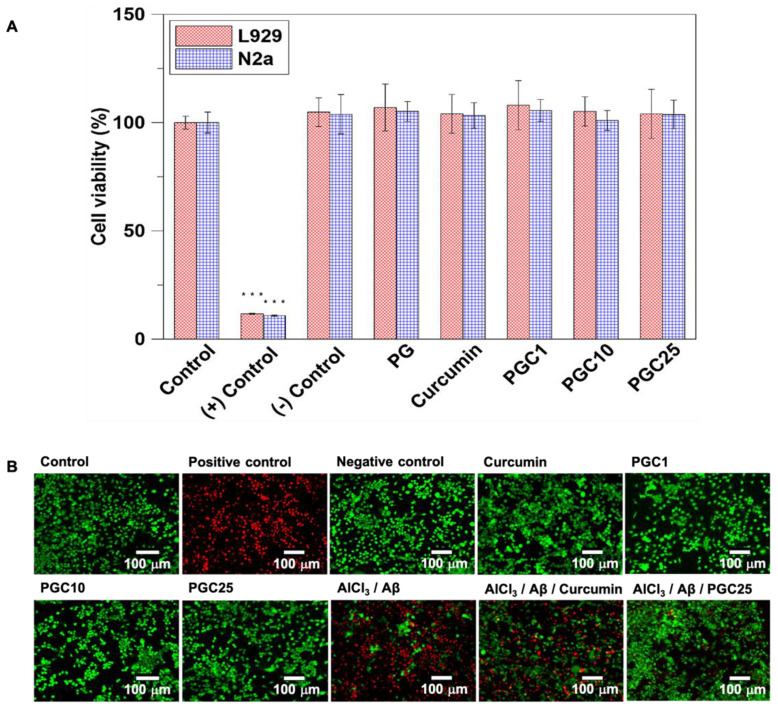
The evaluation of cell viability of thermo-gel and inhibition of Aβ-induced cytotoxicity. (**A**) The cell viability of PG and PGC by WST-1 (*n* = 6, *** *p* < 0.001 compared with control). (**B**) The LIVE/DEAD staining of N2a cells in the control: cells cultured in medium only; Positive control: cells treated with zinc diethyldithiocarbamate; Negative control: cells treated with aluminum oxide; Curcumin: cells treated with 25 μM curcumin; PGC1: cells treated with PGC1; PGC10: cells treated with PGC10; PGC25: cells treated with PGC25; AlCl_3_/Aβ: cells treated with AlCl_3_ and Aβ42; AlCl_3_/Aβ/Curcumin: cells cocultured with AlCl_3_, Aβ42 and 25 μM curcumin; AlCl_3_/Aβ/PGC25: cells cocultured with AlCl_3_, Aβ42, and PGC25.

The biocompatibility of the drug delivery system was determined using the WST-1 assay. Figure 3A indicates cell viability according to the WST-1 assay. There was no significant difference in cell viability for the test groups of PG, 25 μM curcumin, PGC1, PGC10, and PGC25. Based on ISO-10993, we believe that the synthesized PGC would produce no toxicity to the L929 fibroblasts and N2a cells.

The inhibition of the cytotoxic effect by curcumin-free drug and different concentrations of PGC were determined using LIVE/DEAD^™^ staining. As shown in Figure 3B, the dead cells are shown in red and the living cells in green. No significant difference in the ratio of red and green signals among the experimental groups was observed between the control and PGC treatment. We infer that PGC could reduce the cytotoxicity that was caused by the Aβ oligomer. However, the curcumin-free drug only did not significantly reduce the cytotoxicity of Aβ. In the previous study by Gutierres et al. (2015), it indicated that curcumin would degrade in less than an hour [27]. Therefore, this is the reason for the poor inhibition of cytotoxicity in live/dead after three days of co-culture compared to the PGC25.

### 3.3. ThT Stain for Inhibition of Aβ Aggregation

**Figure 4:**
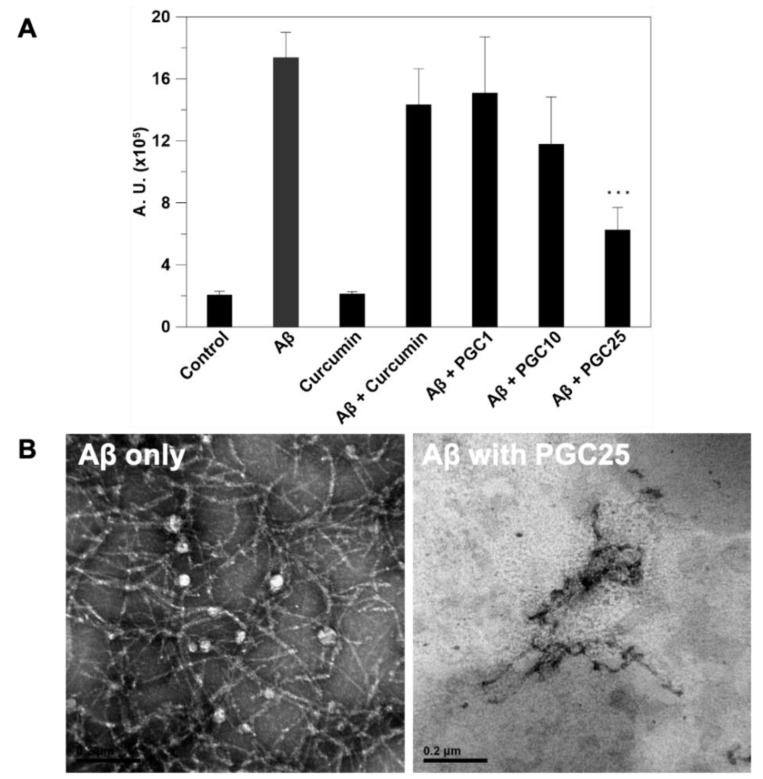
The identification of the inhibition of amyloid fibril aggregation by curcumin and PGC. (**A**) The fluoresce intensity of ThT and (**B**) TEM image of amyloid fibril aggregation (n = 6, *** *p* < 0.001 compared with the control).

The fluorescent dye ThT was used to identify amyloid fibrils. We showed that PGC25 could effectively inhibit Aβ aggregation and prevent Aβ-induced neurotoxicity (Figure 4A). Aβ42 in PBS is known to aggregate and be converted into Aβ fibrils gradually. The Aβ fibrils bind to the ThT dye, resulting in fluorescence emission. The result showed that the emitted fluorescence intensity increased when Aβ42 was present in PBS. However, the PGC25 sharply inhibited the formation of the Aβ fibril or Aβ42 aggregation, therefore, the emitted fluorescence intensity was much lower than that without the addition of PGC25. Only the curcumin-free drug did not inhibit the formation of Aβ fibril or Aβ42 aggregation due to the rapid degradation of curcumin.

Conversely, TEM images showed that curcumin could disassemble amyloid plaques (Figure 4B). Furthermore, the structure of amyloid aggregates became significantly loose with increased drug concentration. The images showed that Aβ incubation with PGC resulted in extensive fragment formation. In the absence of PGC25, the images of Aβ peptides showed dense fibrils. The images were obtained after the co-incubation of Aβ and PGC for three days.

### 3.4. Western Blot Analysis of β-Secretase Inhibition

**Figure 5:**
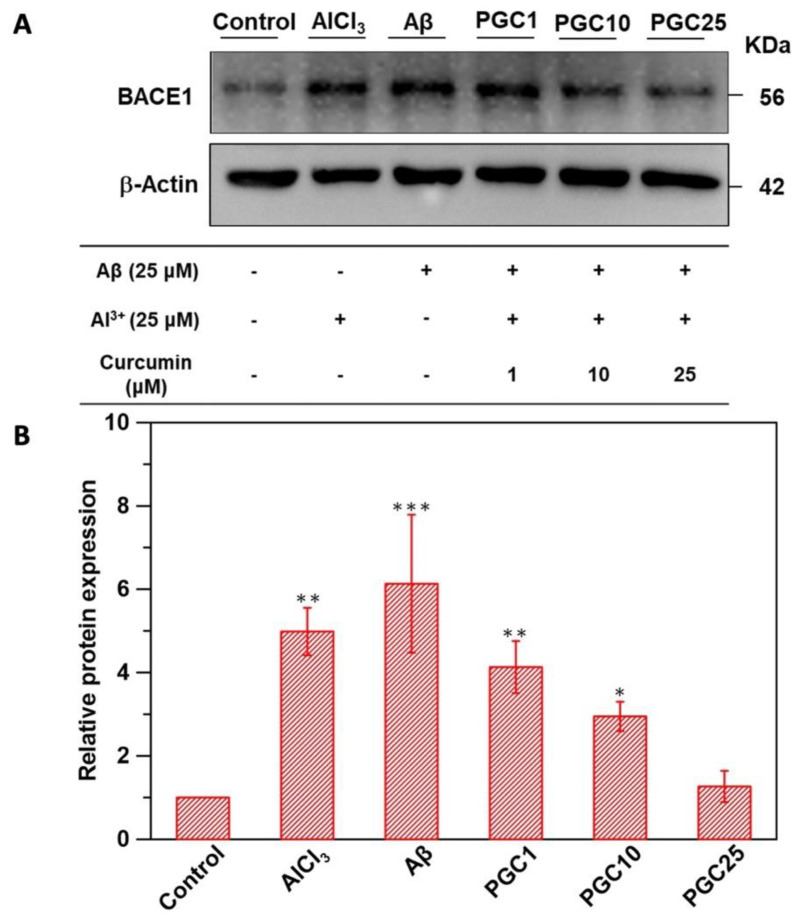
Biochemical analysis. (**A**) The western blot analysis of BACE1. β-actin was used as the loading control. (**B**) The quantitative values of the BACE1 expression data obtained the ratio of BACE1 protein/actin protein band intensities normalized to 1 in the control group (*n* = 6, * *p* < 0.05 compared with control, ** *p* < 0.01 compared with control, *** *p* < 0.001 compared with control).

Western blotting was performed to investigate the BACE1 levels (Figure 5); curcumin was proved to inhibit β-secretase, preventing AD development. The western blot results are presented in Figure 5A. PGC1, PGC10, and PGC25 (final curcumin concentrations of 1, 10, and 25 μM, respectively) significantly inhibited β-secretase, which was induced by Al^3+^ (25 μM) and Aβ (25 μM). We estimated that the PGC25 system could reduce Aβ_1-42_ production and reduce Aβ-induced damage. The measured values of the band intensities are shown in Figure 5B.

### 3.5. Antioxidant Effect and Anti-Inflammatory Effects of PGC

**Figure 6:**
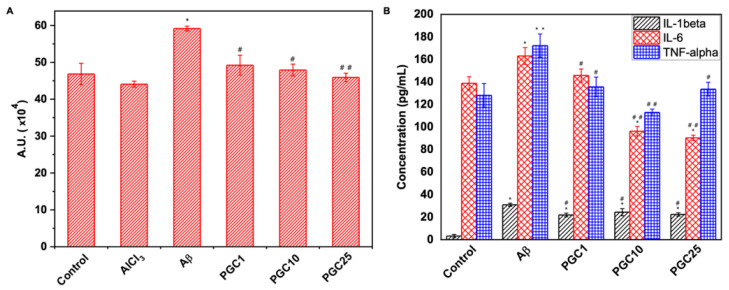
Antioxidant effect and anti-inflammatory effects of PGC. (**A**) Antioxidant activity of PGC. DCFDA was used to measure the intracellular ROS in N2a cells without any treatment (control) and treated with a different concentration PGC (*n* = 6, * *p* < 0.05 compared with control, # *p* < 0.05 compared with Aβ group, ## *p* < 0.01 compared with Aβ group). (**B**) Anti-inflammatory effect of the PGC on Aβ-induced inflammation in BV-2 cells estimated by the expression analysis of inflammation-related genes, TNF-α, IL-6, and IL-1β (*n* = 6, * *p* < 0.05 compared with control, ** *p* < 0.01 compared with control, # *p* < 0.05 compared with Aβ group, ## *p* < 0.01 compared with Aβ group).

The anti-oxidative effects of PGC were determined using the DCFDA assay (Figure 6A). This facilitated the measurement of intracellular reactive oxygen species (ROS) levels in the differentiated N2a cells. DCFDA oxidization generated DCF fluorescence as an indicator of ROS levels. N2a cultured in DMEM was set as the control group and defined as 100%. The ROS level in cells treated with 10 μM Aβ fibrils for 1 h increased by 26% compared to that in the control group. Therefore, Aβ fibrils induced a significant increase in ROS levels. However, the presence of PGC decreased the fluorescence intensity. Meanwhile, there was no significant difference in ROS levels between the control group and the PGC group. These results confirmed that PGC could reduce the ROS levels to the normal level, protecting the neurons from degeneration.

The anti-inflammatory effects of PGC were confirmed using an ELISA kit. The microglial cells BV2 were incubated with Aβ (5 μM) for 6 h to detect IL-1β and for 24 h to detect IL-6 and TNF-α. The results (Figure 6B) showed that PGC1, PGC10, and PGC25 reduced Aβ-induced BV2 cell inflammation. We expected that reducing Aβ-induced inflammation would reduce neuronal degeneration, preventing AD development.

### 3.6. Morris WM Test and Determination of Hippocampal Activity by fMRI

**Figure 7:**
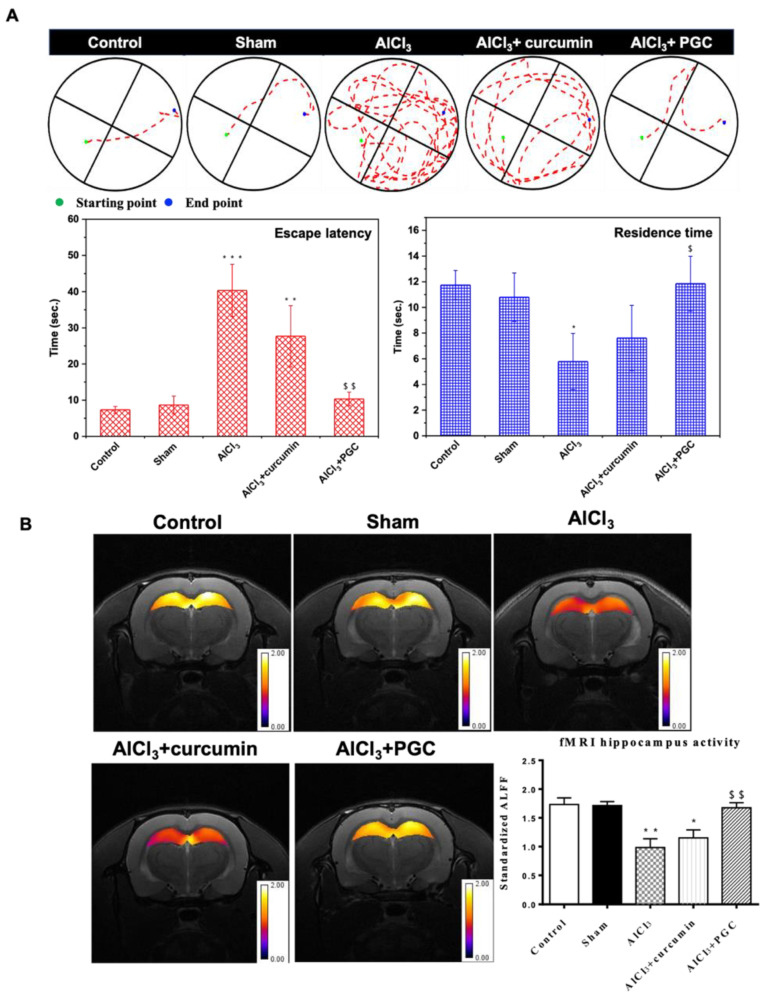
The Morris WM test and determination of hippocampal activity by fMRI. (**A**) The MWM track of the normal rats (control), PBS injection (Sham), AlCl_3_-induced AD rats (AlCl_3_), curcumin-free drug- treated AD rats (AlCl_3_ + curcumin), and PGC treatment (AlCl_3_ + PGC). The starting point (blue) to endpoint (green) of all the tested rats was the same (*n* = 6, * *p* < 0.05 compared with control, ** *p* < 0.01 compared with control, *** *p* < 0.001 compared with control, $ *p* < 0.05 compared with AlCl_3_ group, $$ *p* < 0.01 compared with AlCl_3_ group). (**B**) fMRI analysis of brain activity on rats’ hippocampus. The statistical parametric mapping of the brain activity in the rat hippocampus. The brain activity was determined by the intensity of brightness in the ROI on single shot GRE-EPI images. The intensity of brightness in the ROI of the AlCl_3_ group was about 50% lower than that of the control and AlCl_3_ + PGC group (*n* = 6, * *p* < 0.05 compared with control, ** *p* < 0.01 compared with control compared with control, $$ *p* < 0.01 compared with AlCl_3_ group).

The MWM test was performed to evaluate spatial memory in aluminum-induced AD rats [(+) control]. We administrated aluminum chloride (100 mg/kg, i.p.) three times a week for one month to induce neurodegeneration, which exhibits similar symptoms to AD. We injected a curcumin (35 mg/kg, IM) solution and PGC (curcumin 35 mg/kg, i.m.) to prevent neurodegeneration. The swimming path and escape latency of each group is shown in Figure 7A. The PGC group quickly found the escape platform. Furthermore, upon removing the platform, the PGC group exhibited a longer retention time in the quadrant in which the platform was formerly located. However, the curcumin group could not perform similar to the PGC group. We believe that the curcumin was degraded because of its lack of micelle protection and insolubility in the body environment. Thus, the curcumin PBS solution could not prevent neurodegeneration. The PGC delivery system was shown to enhance the bioavailability of curcumin and prevent neurodegeneration.

Rats were anesthetized and observed by fMRI. T2-weighted RARE images were used for anatomical observations, and single-shot GRE-EPI images were used for brain activity measurements. Coronal MRI brain images of the hippocampus were obtained, and regions of interest (ROI) were selected. Brain activity on the hippocampus was determined by the brightness intensity in the ROI on a single GRE-EPI image (Figure 7B). A coronal MRI image of the hippocampus was taken using functional MRI, and an ROI was selected on the hippocampus. The low-frequency fluctuation amplitude (ALFF) index indicates brain activity in the hippocampus based on ROI analysis. The ALFF index of the AlCl_3_ group was significantly lower than that of the control group, indicating lower brain activity in the hippocampus and more severe AD. After PGC administration, hippocampal activity was significantly increased, with no significant difference compared to the control group.

### 3.7. Histological Analysis and IHC Staining

**Figure 8:**
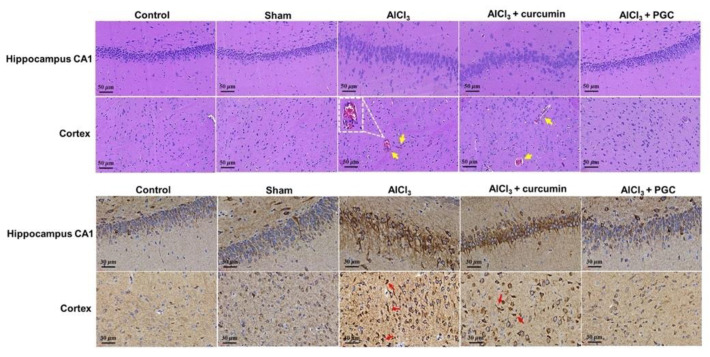
Histological staining. Hematoxylin and eosin (H&E) staining of hippocampal CA1 and cortex regions of the tested rats. Brain sections from rats treated with AlCl_3_ show that pyramidal cells in the hippocampal CA1 region exhibited more severe morphological changes. BACE1 revealed plaque-like staining in the brains of AD rats by immunohistochemistry. Scale bars: 50 and 30 μm.

The morphological changes in the cortex and hippocampus of the rat brain, as indicated by H&E staining, are shown in Figure 8. The brains from rats induced with AlCl_3_ exhibited marked damage in the hippocampus and cerebral cortex compared to the control animals. The most obvious changes were cellular atrophy, shrinkage, cellular necrosis, pyknosis, and deeply stained and dark nuclei (hyperchromatic cells). The nuclei of some cells were pyknotic. Additionally, large cells, which were mostly neuronal swelling and vacuolated cells, were observed. In contrast, cells from hippocampal CA1 and cerebral cortex regions in the control group exhibited a normal morphology. The PGC-treated group presented a substantially lower number of abnormal cells with nuclear condensation. Therefore, PGC protected the brain tissue from Aβ-induced damage.

The increase in the BACE1 level with amyloid load in the AlCl_3_-induced models demonstrated that the BACE1 elevation was triggered by Aβ or amyloid plaques. To initially investigate the relationship of the BACE1 increase to plaques, we stained alternate brain sections from the AlCl_3_ group with BACE1. BACE1-positive accumulations were present in the hippocampus and cortex (Figure 8). However, the Aβ levels in both the cortex and hippocampus were greatly attenuated by PGC prevention, demonstrating that PGC was able to reduce the aggregation and deposition of Aβ.

## 4. Discussion

In this study, we synthesized PLGA-PEG-PLGA triblock copolymers using the ring-opening method to encapsulate curcumin for the prevention of AD. The optimized block copolymer was characterized using ^1^H NMR spectroscopy, which indicated five specific peaks for chemical shifts according to the presence of protons in the PLGA-PEG-PLGA copolymer. For the PLGA copolymer, the peaks at δ 1.50, 4.85, and 5.21 ppm were assigned to methyl, methylene, and methine protons, respectively. Several studies have confirmed that the 4.85 and 5.21 ppm chemical shifts corresponded to GA and LA, respectively [28]. In addition, the PEG methylene proton peak was located at δ 3.53 ppm. According to Yu et al., the LA and GA sequences can be described by distinct peak patterns at 4.85 and 5.21 ppm. The optimized block copolymers followed the intermittent formation of LA and GA sequences, with LA more dominant than GA units [29].

In the FTIR spectrum for the block copolymer (Supplementary Figure S1), the specific vibrations for PEG, LA, and GA shifted to different wavelength numbers (i.e., the C=O ester vibration ranged from 1749.1 to 1747.7 cm^−1^, C–O vibration ranged from 1086.1 to 1083.8 cm^−1^, while the alkyl bending vibration of approximately 1542 cm^−1^ and the O–H stretching vibration at 3468 cm^−1^ corresponded to the end group of PLGA copolymers). Our results regarding the formation of PLGA-PEG-PLGA were consistent with previously reported results [30]. The specific functional groups of the PLGA block copolymer, determined through FTIR, confirmed the structure of the triblock copolymer.

The addition of curcumin helped to form a more robust gel matrix (Supplementary Figure S1). The presence of curcumin resulted in a delay in PLGA interaction and micelle formation. Better polymer hydration occurred with the formation of the high viscosity gel; this was confirmed by the increase in the gel temperature after curcumin incorporation. Similarly, Guo et al. [31] reported the concentration-dependent rheological behavior of methoxyestradiol solid lipid nanoparticles loaded into PLGA-PEG-PLGA thermosensitive hydrogels, accompanied by increased viscosity. In solution, copolymers composed of hydrophilic and hydrophobic moieties can exist either as individual hydrated molecules or as spherical aggregates (Figure 2) [30]. When these copolymers were exposed to aqueous conditions, the hydrophilic PEG and ethylene glycol moieties were oriented to the outside of the micelles, coming in contact with water. In contrast, the hydrophobic PLGA moieties were located inside the micelles due to their hydrophobic intermolecular interactions. Thus, self-assembled micelles were formed [32].

A previous study described the following three mechanisms controlling drug release from PLGA matrices (Figure 2) as: (i) Fickian diffusion through the polymer matrix; (ii) diffusion through water-filled pores (water channels) by permeation of water into the matrix; and (iii) release by erosion of the polymer matrix [33]. The actual drug release from the polymer matrix can be controlled by combining these three mechanisms [34]. Most drugs used in AD treatment are designed to be highly hydrophobic because the target area is usually located inside the brain, therefore, these drugs are designed to pass through the blood–brain barrier (BBB). Only hydrophobic and small molecule drugs can pass through the BBB via the blood circulation [35,36]. In this study, PGC showed excellent anti-inflammatory and antioxidant properties and modulation of microglia (Figure 3, Figure 4, Figure 5 and Figure 6). The in vitro study results suggest that PGC could inhibit the Aβ aggregation and inflammatory responses of the microglia.

The fluorescent dye ThT has been widely used to identify amyloid fibrils. Fluorescence imaging showed that PGC could inhibit amyloid aggregation. The ALFF index of the AlCl_3_ group was significantly lower than that of the control group, which indicates lower brain activity in the hippocampus and more severe AD. After PGC prevention, the hippocampus activity increased significantly and exhibited no significant difference compared with the control group (Figure 7). Compared with the control rats, Alzheimer’s disease induced (ADI) rats showed significant differences in escape latency, indicating that AlCl_3_ injection can effectively induce learning deficits. In contrast, the escape latency of the PGC rats was significantly shorter than that of the ADI rats, and there was no significant difference in learning behavior compared with the control rats, indicating a significant improvement in the performance of the rats after PGC administration (Figure 7). In addition, ADI rats performed more poorly in the on-platform search. In contrast, control and PGC rats exhibited a focused search strategy with the shortest swim path and longest dwell time in the quadrant where the platform was initially placed.

According to histological analysis and IHC staining (Figure 8), the brains of AlCl_3_-induced rats exhibited considerable damage in the hippocampus and cerebral cortex compared to that of the control animals. In contrast, the PGC-treated group exhibited substantially lesser abnormal cells with nuclear condensation. The increase in the BACE1 level with amyloid load in the AlCl_3_-induced models demonstrated that the BACE1 elevation was triggered by Aβ or amyloid plaques. However, the Aβ levels in both the cortex and hippocampus were greatly attenuated by PGC prevention, demonstrating that PGC was able to reduce the aggregation and deposition of Aβ. Furthermore, there was no significant difference in viability between the cells treated with PG and PGC (Figure 3). As a biomaterial, PLGA-PEG-PLGA, widely used for tissue repair, is biocompatible and non-toxic to the human body [37,38].

In this study, we used AlCl_3_ to induce rats with AD. The multiple neurotoxic effects of aluminum led to the impaired clearance of Aβ42 peptides that drive amyloidogenesis and AD-type changes. Aluminum induces NF-kB [39] and upregulates micro RNAs (miRNAs) in intracellular and intranuclear compartments [40], and downregulates the key microglial intramembrane phagocytosis sensor protein triggering receptor expressed on myeloid cells 2 (TREM2) [41]. Lack of sufficient TREM2 impairs microglia-mediated phagocytosis and clearance of Aβ42 peptide monomers. TREM2 deficiency (but not the TREM2-associated TYROBP/DAP12 adaptor protein required for phagocytosis and Aβ42 peptide phagocytosis) has been widely reported in AD brains and in stressed microglia [42]. In the extracellular space, aluminum aggregates Aβ42 peptide monomers into dense insoluble spherical clumps and promotes the formation of senile plaques. The movement of Al^3+^ across the plasma membrane is unclear but may involve both active and passive transport. Although microglia are capable of phagocytosing Aβ42 peptide monomers, they may have difficulty expelling higher-order aggregates, leading to microglial activation and pathogenic pro-inflammatory responses that contribute to AD neuropathology.

Natural products have been used to delay disease progression in elderly and AD patients. Several research groups have investigated the efficacy of natural products and antioxidants including vitamin E, curcumin, ginkgo biloba, and melatonin to determine whether antioxidants can reduce Aβ and tau lesions and enhance cognitive function in mouse models of AD [43,44,45,46]. Tang and Taghiglou [10] focused on the mechanism of action of curcumin in AD including its inhibitory effect on Aβ and tau, copper-binding ability, cholesterol-lowering ability, anti-inflammatory activity, regulation of microglia, AChE inhibition, oxidative and anti-inflammatory properties, and modification of insulin signaling pathways. Wang and colleagues investigated the anti-BACE-1 and behavioral activities of curcumin obtained from C. longa rhizomes. Their results suggest that structural features such as saturation, type of carbon backbone and functional groups, and hydrophobicity appear to play a role in determining the potency of BACE-1 inhibition by curcumin [47].

The results of these studies were positive and consistent with our findings; antioxidant treatment showed reductions in soluble Aβ levels and improvements in cognitive behavior. An in vitro study reported that curcumin inhibited Aβ aggregation and induced its depolymerization to form fibrillar Aβ40 [10]. Several in vivo studies have shown that curcumin promotes the breakdown of existing amyloid deposits, prevents the aggregation of new amyloid deposits, and even reduces the size of remaining deposits [48]. Furthermore, curcumin and its derivatives inhibit the formation of fibrillar Aβ from Aβ monomers in vitro and destabilize pre-formed fibrillar Aβ, suggesting that curcumin can prevent Aβ toxicity [49]. Levels of Aβ and Aβ deposits were reduced in the brains of APP mice treated with low-dose curcumin compared to the brains of untreated APP mice. At higher concentrations, curcumin binds to Aβ and blocks its self-assembly [10]. A recent study reported that curcumin disrupts Aβ40 and Aβ42 [50].

Furthermore, curcumin-derived isoxazoles and pyrazoles bind to Aβ and inhibit AβPP metabolism [51]. Curcumin protects PC12 cells and normal human umbilical cord endothelial cells from Aβ-induced oxidative stress [52]. Curcumin reduced the levels of oxidized proteins and IL1B in the brains of APP mice [53]. Moreover, it enhances Aβ uptake by macrophages in AD patients; bone-marrow-derived dendritic cells can correct the immunodeficiency of AD patients and may be used as immunotherapy [54]. Curcumin also inhibits peroxidase and modulates cytopathology in AD patients [55], binds to the redox-active metals iron and copper, and inhibits inflammatory damage by preventing metal-induced NF-kB overexpression [56].

Oxidative stress and neuroinflammation are the two main factors involved in the progression of neurodegenerative diseases. Therefore, compounds with antioxidant and anti-inflammatory properties can be used to provide significant neuroprotection. Overall, antioxidant treatments result in an effective outcome and delays disease progression in elderly individuals; however, it is less effective in patients with severe AD. In this study, AD development could be prevented in rats by administering a PLGA-PEG-PLGA curcumin delivery system via IM injection. However, the prevention against AD should be achieved by a non-invasive method. Therefore, future research should be performed on the non-invasive methods that can prevent AD.

This study had some limitations. Since curcumin has been reported to sequester copper ions, it is not clear if a rat model of Alzheimer’s disease employing aluminum salt injections is the appropriate experimental paradigm to test the potential neuroprotective activity of curcumin. The observed behavioral effects of the PLGA-PEG-PLGA curcumin formulation used in the present study could simply be caused by the binding of curcumin to the aluminum.

## 5. Conclusions

Current AD treatments focus on patients in whom AD has already developed; the treatment results are not highly promising in these patients. We believe that prevention of AD onset might be the best approach to avoid high-dose AD treatment with low effectiveness. We developed a PLGA-PEG-PLGA curcumin (PGC) delivery system in this study. PGC exhibited good biocompatibility and biodegradability. The system presented sol–gel behavior and sustained the release of curcumin-loaded micelle for 20 days. The effects of curcumin would protect neurons from degeneration, along with multi-target treatment. The curcumin-loaded micelles reduced oxidative stress and cytotoxicity in N2a cells and inhibited inflammation in BV2 cells. Additionally, PGC was able to prevent neurodegeneration in an aluminum-induced animal model. We delivered one shot a month of PGC by IM injection, and the MWM test and histological staining proved that the designed PGC could prevent AD development in rats. In the future, this approach may enable patients with AD to overcome the aforementioned obstacles in their treatment.

## Figures and Tables

**Table 1:** The groups of animals used in animal study.

Group	Treatment
1 (control group)	Rats without any treatment
2 (sham group)	Rats treated with PBS (i.p.)
3 (AlCl_3_)	Rats induced with AlCl_3_ (100 mg/kg, i.p.) three times a week
4 (AlCl_3_ + curcumin)	Rats induced with AlCl_3_ (100 mg/kg, i.p.) three times a week and curcumin-free drug (16.5 mg/kg, i.m.) every four weeks
5 (AlCl_3_ + PGC25)	Rats induced with AlCl_3_ (100 mg/kg, i.p.) three times a week and curcumin (16.5 mg/kg, i.m.)-loaded with micelle solution every four weeks

## Data Availability

The datasets used and/or analyzed in the current study are available from the corresponding author on reasonable request. Data is contained within the article and Supplementary Materials.

## Supplementary Materials

The following supporting information can be downloaded at: https://www.mdpi.com/article/10.3390/antiox11040727/s1, Figure S1: The characterization of triblock copolymer and the gelation identification of copolymer.
