# Supplementary material for: Investigating a Curcumin-Loaded PLGA-PEG-PLGA Thermo-Sensitive Hydrogel for the Prevention of Alzheimer’s Disease
Source: Antioxidants (Basel). 2022 Apr 7;11(4):727. doi: 10.3390/antiox11040727 (PMC9026862; doi:10.3390/antiox11040727)
Supplement: Supplementary file 1 [file antioxidants-11-00727-s001.zip › antioxidants-1639869-Supplementary.pdf]

Supplementary Data

Characterization of triblock copolymer

The ^{1}H NMR spectrum of the copolymer is shown in Supplementary Figure 1A; the chemical shifts for the copolymer were δ 1.55 ppm (CH_3 of LA), δ 3.65 ppm (CH_2 of PEG), δ 4.20 ppm (CH_2 of PEG), δ 4.80 ppm (CH_2 of GA), and δ 5.20 ppm (CH of LA). The ratio of LA and GA was 3:1.

The FTIR spectrum of the copolymer is shown in Supplementary Figure 1B; the band at 1750 cm^{-1} (C=O, stretching) was considerably stronger in the copolymer than in PEG because of the PLGA that was introduced into the copolymer. The band at 3497 cm^{-1} (-OH, stretching) for the LA or GA structure in the polymer product and the band at 1189 cm^{-1} (C-O-C, stretching) for the linkage of ester in the polymer proved that LA and GA were inducted into PEG and that PLGA-PEG-PLGA was synthesized.

The molecular weight was determined by GPC. The number-average molecular weight (Mn), weight-average molecular weight (Mw), and polydispersity (PD) were 3738, 4468, and 1.2, respectively. The low PDI of the copolymer confirmed its formation and purity.

The sol-gel behavior is presented in Supplementary Figure 1C. The aqueous solution was transformed to gel when the micelle concentration was above 20%. We used a 20% micelle solution to conduct further experiments in this study. Supplementary Figure 1D depicts blank and curcumin-loaded micelle solutions at different temperatures. The solution transforms into the gel rapidly. The gelation temperature was confirmed using the rheometer. The elastic modulus (G’) and viscous modulus (G’’) were measured, and the gelation temperature was confirmed when G’ was greater than G’’. Results of the rheological studies are presented in Supplementary Figure 1E and Supplementary Figure 1F; the gelation temperature of the curcumin-loaded micelle solution was lower than that of the blank micelle solution. We estimated that curcumin loading caused the change in gelation temperature because the curcumin-loaded micelles exhibited a smaller particle size than the blank micelles. Thus, the intermolecular force would be stronger, and particles aggregate more easily to form the gel.

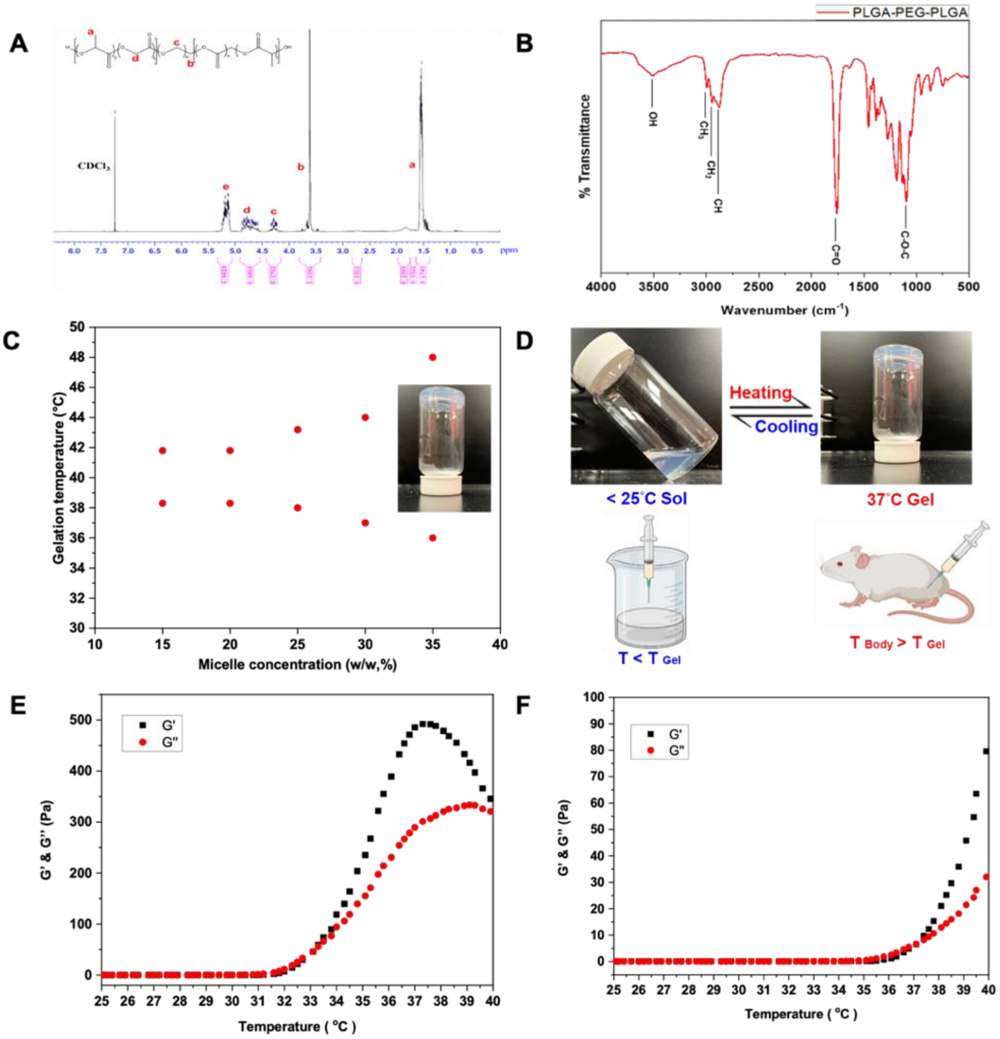

Supplementary Figure S1. The characterization of triblock copolymer and the gelation identification of copolymer. (A) 1H NMR spectrum of the copolymer, (B) the FTIR spectra of the copolymer, (C) the sol-gel behavior of copolymer, (D) photograph of sol-gel behavior in different temperature, (E) the rheometer result of PG and (F) PGC.
